# The impact of comorbidities on the prognosis of patients with septic arthritis

**DOI:** 10.3389/fmed.2025.1567229

**Published:** 2025-06-18

**Authors:** Muhammad Umair Akhtar, Zaid Chilmeran, Abu-Baker Khalid Sharafeldin, Yahya Mostafa Waly, Mustafa Tariq Khan, Salim Fredericks

**Affiliations:** School of Medicine, Royal College of Surgeons in Ireland–Bahrain, Al-Muharraq, Bahrain

**Keywords:** septic arthritis, comorbidities, prognosis, mortality, treatment outcomes

## Abstract

Septic arthritis is a serious infection that can lead to joint destruction, sepsis, and high mortality rates, particularly in elderly patients and those with comorbid conditions. Comorbidities such as diabetes, rheumatoid arthritis, chronic kidney disease, and liver disease can complicate the diagnosis, treatment, and overall prognosis of the disease. These conditions may impair immune function, delay diagnosis, and hinder effective antimicrobial therapy, thereby increasing the risk of severe complications and poor outcomes. This review explores the impact of comorbidities on the prognosis of patients with septic arthritis, emphasizing the need for tailored management strategies to improve outcomes in this vulnerable population. Understanding the interplay between comorbid conditions and septic arthritis is essential for optimizing treatment approaches and enhancing patient care.

## 1 Introduction

Septic arthritis (SA) is a potentially life-threatening condition characterized by the acute infection of a joint, typically resulting in inflammation, pain, and impaired function (1–3). It can rapidly progress to joint destruction and systemic spread, making prompt diagnosis and treatment critical for improving patient outcomes (4). The pathophysiology of SA involves the hematogenous spread of pathogens, which leads to the invasion and inflammation of the synovium (5). *Staphylococcus aureus*, including methicillin-resistant *Staphylococcus aureus* (MRSA), is the most common cause of SA, with *Streptococcus pneumoniae*, *Enterococcus faecalis*, and *Pseudomonas aeruginosa* also identified as causative agents (6). These microorganisms induce a complex inflammatory response (1–3) that not only compromises the affected joint but can also precipitate sepsis (7), further complicating the clinical picture.

Elderly patients with SA face high mortality rates, often associated with comorbidities such as diabetes, preexisting joint disease, and challenges in early diagnosis, even with aggressive management (8). With recent studies providing updates on SA, management strategies have become clearer, aiding in prompt diagnosis and the initiation of early antibiotic therapy (9). Despite this, SA remains associated with significant morbidity and mortality, particularly among vulnerable populations (10). The risk of SA increases with age, especially in individuals with compromised immune systems or chronic conditions (4, 11, 12). In-hospital mortality rates for SA range from 7% to 15% (13), and long-term complications, such as joint destruction and functional impairment (14), further highlight its impact as a critical concern in both clinical and public health contexts.

Comorbidities, defined as the “co-occurrence of distinct diseases” alongside the primary disease (15), have long been recognized as important factors in determining the prognosis, treatment outcomes, and mortality of patients with SA (16). Chronic conditions such as diabetes mellitus, rheumatoid arthritis, and cardiovascular disease, among others, can complicate both the diagnosis and management of SA (17, 18). Understanding the interplay between these comorbid conditions and the outcomes of SA is crucial for improving patient management and outcomes, as they influence both the course of the infection and the patient’s long-term prognosis, ultimately affecting their ability to recover.

## 2 Comorbidities in septic arthritis

### 2.1 Diabetes mellitus

Diabetes mellitus (DM) is a common comorbidity and one of the main risk factors for SA (19), significantly increasing the risk of mortality in these patients (10). Studies show that DM raises the risk of SA by a factor of 3.3 (12). The increased susceptibility to infections in diabetic patients can be attributed to multiple factors, including poor glycemic control (20), chronic low-grade inflammation (inflammaging) (21), and other diabetes-related pathologies (22), all of which impair the immune system and make patients more prone to infections like SA.

Hyperglycemia impairs polymorphonuclear cell function by reducing leukocyte mobilization, causing chemotaxis defects, and limiting energy for pathogen uptake. Diabetes also reduces intracellular killing of microorganisms and, through diabetic neuropathy, can increase the risk of SA by promoting skin infections (12).

Diabetic patients are statistically significantly more likely to experience treatment failure (23), hence, necessitating longer hospitalization durations (24) and additional interventions due to the cases being complicated (25, 26). Additionally, diabetes is significantly associated with infections caused by multi-drug resistant organisms (MDROs), including MRSA, which complicates antibiotic management and often necessitates the use of more potent, nephrotoxic agents (27, 28).

Although direct studies on SA are limited, potential mechanisms by which diabetes may affect its outcomes are multifactorial. Hyperglycemia impairs the function of neutrophils, macrophages, and other immune cells, reducing their ability to kill pathogens effectively (29, 30). Chronic hyperglycemia also causes glycosylation of immune proteins, further weakening host defenses (30). Furthermore, vascular endothelial dysfunction in diabetic patients leads to reduced blood flow and oxygen delivery to affected joints, delaying tissue repair and increasing the risk of persistent infection (31). Diabetes also promotes a state of chronic low-grade inflammation, which can increase susceptibility to infections, and hinder its resolution (32). Low-grade inflammation is characterized by elevated levels of pro-inflammatory cytokines such as IL-6 and TNF-α, and these cytokines can serve as markers for the presence and progression of arthropathy in individuals with DM (33–35).

In addition to these pathogenic mechanisms, practical management modifications are essential to optimize outcomes in diabetic patients with SA. Specifically, a recent network meta-analysis found that a blood glucose target range of 110–144 mg/dL was best for reducing infection risk in critically ill patients (36). Furthermore, early and close involvement of interdisciplinary services, such as endocrinology, is recommended to individualize glycemic management strategies, thereby enhancing infection control and overall outcomes (37).

### 2.2 Immunosuppression

Immunosuppressed individuals, including those with HIV, malignancy, or those receiving steroid therapy, are at significantly higher risk for developing infections, such as SA (38–41). HIV impairs the immune response by driving persistent viral replication that leads to CD4+ T-cell depletion, immune dysregulation, and functional defects in both cellular and humoral immunity, thereby increasing susceptibility to opportunistic infections (42).

In cancer, tumor growth induces peripheral immune cell reorganization, resulting in systemic immunosuppression. These systemic immune changes also impact the efficacy of immunotherapies, complicating treatment outcomes (40).

Glucocorticosteroids suppress inflammation by preventing neutrophils and monocytes from reaching inflammatory sites, leading to neutrophilic leukocytosis, eosinopenia, monocytopenia, and lymphocytopenia (43). Prolonged use of corticosteroids increases the risk of opportunistic infections due to their immunosuppressive effects (41).

In addition, immunocompromised patients may be infected by less common organisms. The signs and symptoms of SA in these patients are often more subtle, and serum inflammatory markers may not be elevated, making diagnosis more challenging. Furthermore, the synovial fluid leukocyte count and percentage of neutrophils, which are helpful in predicting bacterial septic arthritis, may be lower in immunocompromised patients compared to immunocompetent patients (44).

The prognosis for SA in immunosuppressed patients is generally poorer, with higher rates of morbidity and mortality (17). Immunosuppressed individuals, especially those receiving large doses of corticosteroids or other immunosuppressive agents, are extremely susceptible to infections and have a poorer ability to recover from established infections (38, 41, 42, 45). Additionally, penetrating trauma, such as local corticosteroid therapy, can lead to SA in atypical joints (46). This underscores the importance of prevention in managing these conditions, particularly for transplant recipients who face a heightened risk of infections (47), as well as cautiously administering intraarticular corticosteroid injections as they increase the risk of infection in these patients (12, 48, 49).

### 2.3 Chronic kidney disease

Patients with chronic kidney disease (CKD) are at a heightened risk of developing infections, including SA, due to uremic immune dysfunction and frequent hospitalizations (50). These factors contribute to a weakened immune response, making it easier for infections to establish and progress.

The prevalence of SA among patients with end-stage renal disease (ESRD) is notably high, 50 times greater than the general population. A study analyzing data from the US Renal Data System identified 7,009 cases of SA, corresponding to an incidence of 514.8 per 100,000 person-years (51).

CKD predisposes individuals to infections such as SA through multiple mechanisms, largely driven by uremic immune dysfunction and altered drug metabolism. Uremic toxins inhibit drug transporters and reduce the activity of cytochrome P450 enzymes, affecting both Phase I (cytochrome P450) and Phase II (glucuronidation, acetylation) metabolic reactions (52, 53). CKD also alters protein binding, drug elimination, and the volume of distribution, complicating the management of infections (53). Efflux and uptake transporters, such as p-glycoprotein and organic anion and cation transporters, are disrupted, impairing the metabolism of both endogenous substrates like hormones, vitamin D and fatty acids, as well as exogenous drugs (53). Furthermore, CKD selectively modulates hepatic CYP enzyme activity, with transcriptional and posttranslational changes induced by uremic toxins further exacerbating these effects (54).

In addition to metabolic changes, uremia contributes to immune dysfunction by impairing leukocyte activity, including lymphocytes, monocytes, neutrophils, and dendritic cells (46). The accumulation of inflammatory cytokines, resulting from decreased renal clearance, further suppresses immune responses and promotes a pro-inflammatory state (55). These immune deficits, combined with CKD-induced delays in tissue recovery and wound healing, significantly increase susceptibility to infections such as SA and complicate resolution (56).

In managing SA in patients with CKD, careful antibiotic selection and dosing are essential to balance efficacy against the causative pathogen and the potential for nephrotoxicity. Dosages should be adjusted based on renal function, typically using creatinine clearance or glomerular filtration rate to guide dosing, to prevent drug accumulation and reduce the risk of adverse effects. A study on SA in end-stage renal disease patients emphasizes the importance of appropriate antibiotic therapy in this population (51). For instance, in cases of SA due to MRSA, vancomycin is frequently employed. In CKD patients, however, dosing must be individualized. According to one guideline, vancomycin should be dosed every 8–12 hours through levels of 15–20 (10) mg/L for severe infections such as SA, with dose adjustments made based on renal function and therapeutic drug monitoring (57). However, prolonged use of aminoglycosides must be avoided due to the increased risk of nephrotoxicity, especially in elderly patients. In both cases of vancomycin or aminoglycosides, plasma drug levels should be monitored due to their appreciable renal clearance (4).

Furthermore, regular monitoring of renal function is crucial during antibiotic therapy. This includes assessing serum creatinine levels, urine output, and electrolytes to detect any deterioration in kidney function promptly. The association between CKD and adverse outcomes in SA highlights the need for vigilant monitoring (58).

### 2.4 Rheumatoid arthritis and inflammatory arthropathies

Patients with rheumatoid arthritis (RA) face a significantly increased risk of SA due to joint damage, poor skin condition, and immunosuppression, with studies showing that RA is the most common pre-existing joint disease associated with SA (4, 12, 59, 60). Approximately 20% of patients with SA have RA, despite the condition only affecting 1% of the general adult population, implying an odds ratio of 20 (12). The chronic synovitis and abnormal joint structures characteristic of RA create an environment conducive to bacterial survival and growth, further elevating the risk (61, 62). Additionally, the immunosuppressive treatments commonly used in RA management, including intra-articular corticosteroids, disease-modifying antirheumatic drugs (DMARDs), and biologics such as anti-TNF agents, further suppress immune function, predisposing patients to infections (44, 49, 63, 64).

Temporarily halting or adjusting the dosage of immunosuppressive medications may be necessary during active infections to enhance the body’s ability to combat pathogens. For example, anti-TNF agents should be stopped until the infection clears, as they carry about twice the risk of serious infection compared to methotrexate, especially in the first six months of treatment. Methotrexate may be continued or adjusted depending on how severe the infection is, with guidance from a rheumatologist (65). However, such decisions should be individualized and made in consultation with a rheumatologist.

The prognosis of SA in RA patients is often poor, with delayed diagnosis being a major challenge. The clinical features of SA frequently overlap with those of RA, particularly in patients who have both conditions, leading to diagnostic delays (66, 67). As a result, these patients are more likely to experience joint destruction and poor functional outcomes (68–70), as well as overlapping symptoms found in both diseases, such as joint pain, swelling, tenderness, warmth, and limited range of motion, making differentiation difficult (13, 59, 71, 72). Furthermore, the presence of RA is associated with poor outcomes and significantly increases mortality and morbidity rates in patients with SA, emphasizing the severity of this condition in this population (3, 10, 59, 60, 73). It is important to note that RA is a distinct clinical entity from non-rheumatologic causes of immunosuppression (e.g., malignancy, HIV), although both are relevant in SA.

### 2.5 Liver disease

One of the most common diseases associated with the hepatobiliary system is chronic liver disease (CLD), which has also been recognized as one of the leading factors of worldwide mortality and morbidity (74, 75). Studies have shown that patients with liver disease, particularly those with cirrhosis, have a 1.8 times higher risk of developing SA (76). One of the underlying mechanisms behind this entails impaired immune function which can arise in conditions like Cirrhosis associated immune dysfunction (CAID) (77). This is due to the liver’s function in producing acute-phase reactants aiding in immune regulation, hence damage to the liver could lead to an increase in SA susceptibility.

Another causative factor is decreased phagocytosis which is caused by the liver’s impaired function of neutrophils and macrophages, therefore leading to increased susceptibility to infection (78). Moreover, the liver is involved in the production of clotting factors; hence, liver disease would lead to coagulopathy, resulting in joint effusions and bleeding, mimicking the signs of SA (79).

There are three specific liver diseases that have been associated with an increased susceptibility to SA, including alcoholic liver disease, viral hepatitis (Hepatitis B or C), and cirrhosis (74). Chronic consumption of alcohol has been illustrated to impair adaptive and innate immunity, therefore increasing the susceptibility to bacterial infections (80). Moreover, studies show that Hepatitis B and C lead to alterations in the immune system, rendering individuals immunocompromised thus increasing the vulnerability to infections (81). Furthermore, patients with cirrhosis frequently develop spontaneous bacterial peritonitis (SBP) which often coexists with bacteremia leading to SA (82).

CAID is another possible mechanism leading to SA in liver disease patients. CAID causes low-grade systemic inflammation secondary to reduced ability of the liver to clear toxins as well as episodic activation of immune cells as a result of the release of damage associated molecular patterns released from necrotic liver cells (83). This chronic inflammation, combined with the liver’s reduced ability to produce complement proteins and impaired local immune surveillance, makes patients with liver disease more susceptible to infections, including SA.

Due to the aforementioned mechanisms, patients with both liver disease and SA have been shown to present atypically. Due to the immune dysfunction, patients would present with mild or no fever, alongside signs of liver disease including ascites, encephalopathy, and peripheral edema (18, 84).

## 3 Discussion

**FIGURE 1:**
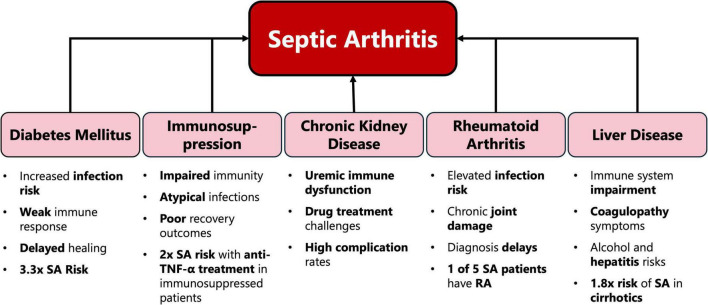
Overview of main comorbidities in patients with septic arthritis.

The results of our review demonstrate that multimorbidity significantly complicates the clinical management of SA and worsens patient outcomes through various pathways (Figure 1 and Table 1). The interplay between these comorbid conditions can create a synergistic effect, leading to heightened systemic inflammation, impaired immune response, and reduced physiological resilience. This synergy often exacerbates the severity of SA, delays recovery, and increases the likelihood of long-term functional impairment.

**TABLE 1:** Pathophysiological mechanisms and associated comorbidities in septic arthritis.

Pathophysiological mechanism	Associated comorbidities
Immune dysfunction	Diabetes mellitus, immunosuppression, chronic kidney disease, rheumatoid arthritis, liver disease
Chronic inflammation	Diabetes mellitus, chronic kidney disease, rheumatoid arthritis, liver disease
Delayed wound healing and vascular dysfunction	Diabetes mellitus, chronic kidney disease, liver disease
Altered drug metabolism	Chronic kidney disease, liver disease
Increased susceptibility to MDROs	Diabetes mellitus, immunosuppression, chronic kidney disease, liver disease
Atypical presentation and diagnostic delays	Chronic kidney disease, rheumatoid arthritis, liver disease

### 3.1 Multimorbidity and its impact

The Charlson Comorbidity Index (CCI) is a widely used tool for quantifying the burden of multimorbidity and predicting patient prognosis (85). Originally developed to estimate 10-year survival in patients with chronic diseases, the CCI assigns weighted scores to various comorbid conditions based on their impact on mortality risk (86). It has been demonstrated that higher CCI scores correlate with poorer outcomes in patients with SA. For example, the findings of one study revealed that patients who died due to SA had a median age-adjusted CCI of 8, suggesting a significant correlation between higher CCI scores and increased mortality (14).

The presence of multiple comorbidities also poses diagnostic and therapeutic challenges. Comorbid conditions like diabetes and CKD can mask early signs of SA or complicate its clinical presentation (87). Furthermore, these conditions often necessitate tailored therapeutic approaches. Patients with CKD may require adjustments to antibiotic dosing due to altered pharmacokinetics, while those with cardiovascular comorbidities may face increased perioperative risks during joint debridement or replacement surgeries.

### 3.2 Personalized treatment plans

As a result of these comorbidities, patients would require adjustments in the treatment regimens to optimize outcomes due to the underlying conditions that need to be taken into account. In the case of diabetic patients suffering from SA, some have been observed to insufficiently respond to standard treatment and therefore require surgical intervention for drainage and debridement of the joint (25). Diabetic patients are additionally more susceptible to MDRO infections, meaning the antibiotic regimen used would have to be altered to possibly more nephrotoxic, however, potent drugs (27, 28). With regards to immunosuppressed patients, gram-negative coverage such as a third-generation cephalosporin, should also be considered (18). Patients with SA suffering from CKD must undergo precise dose leveling and monitoring when administering nephrotoxic drugs used in the treatment of SA such as Vancomycin in gram-positive cocci arthritis, or Piperacillin/tazobactam in gram-negative rod arthritis, to prevent further nephrotoxicity and acute kidney injury (18, 88, 89). Certain RA patients fall under the immunosuppressed group when managing for SA. This is secondary to further usage of the immunosuppressive medications such as intra-articular corticosteroids and disease-modifying antirheumatic drugs suppressing the immune system; consequently, gram-negative coverage should also be considered in these patients (90).

### 3.3 Multidisciplinary management

The effective management of SA requires a collaborative approach involving multiple specialists to optimize patient outcomes. Rheumatologists play a crucial role in early diagnosis and management of SA by utilizing microscopic analysis and synovial fluid culture as fundamental diagnostic tools (91). Endocrinologists contribute by managing metabolic disorders such as diabetes, a known risk factor for SA (4), helping to prevent and mitigate complications. Nephrologists are essential in managing cases among patients with end-stage renal disease (ESRD), who face a significantly higher risk of developing SA due to immune dysfunction from uremia and chronic vascular access (51).

Additionally, infectious disease specialists (IDS) play a key role in selecting appropriate antimicrobial therapy, ensuring effective infection control, and monitoring for complications such as antibiotic resistance. Their expertise is crucial in managing infections, optimizing antibiotic use, and preventing the emergence of resistant strains, ultimately improving patient outcomes (92).

Healthcare providers can develop comprehensive, patient-centered treatment plans that address both the infection and underlying comorbidities by integrating expertise across these disciplines. Importantly, including orthopedic surgeons in the care team is essential. Surgery may be required in cases where response to conservative medical therapy is poor (18). Similarly, for severe cases, surgical intervention may be required from the beginning.

### 3.4 Gaps in knowledge and future directions

Sickle cell disease (SCD), an inherited hematological abnormality in hemoglobin leading to alteration of RBC shape into a sickled shape, was found to complicate into SA especially in children. For instance, one in 500 African–American infants born in the US are affected by SCD, including the trait and other variants, with an estimate of up to 100,000 patients living in the United States (93). In patients suffering from SCD, there is systemic inflammation and oxidative stress leading to poor bacterial opsonization, increasing the susceptibility of those patients to developing SA as well as osteomyelitis which could lead to osteonecrosis (94). Another proposed mechanism is the increased susceptibility in vaso-occlusion of the blood supply, as leukocytes, particularly neutrophils, adhere abnormally to endothelial cells (95), and sickle erythrocytes adhere to these immobilized leukocytes in the endothelium (96). This leads to a slower blood flow and promotes sickle RBC sickling (95), thus, causing microvascular occlusion, vaso-occlusive crisis, and tissue ischemia (96). Recurrent and repeated vaso-occlusion causes chronic disabling arthritis (96), as well as fibrosis and progressive atrophy of the spleen (autosplenectomy) (97). This leads to hyposplenism and increases the susceptibility of sickle cell anemia children to infection with encapsulated bacteria (97), such as *Staphylococcus aureus* and the *Streptococcus* species (98, 99), which are the most common causes of SA (18).

However, currently there is a lack trials and literature discussing epidemiology, management, prognosis or natural history of SA in SCD patients (100). This emphasizes the need for large, multi-center, longitudinal cohort studies to discuss the epidemiology behind this relationship and further analyze the mortality rates to determine the significance of this correlation. Randomized controlled trials and other trials are needed to explore optimum management plans in these groups of patients. Primary factors contributing to the scarcity of large cohort studies and trials done on specific comorbid groups can arise simply due to the lack of identifying specific comorbid patients suffering from SA at any simultaneous time. Another factor may stem from the idea that these patients tend to have other underlying comorbid diseases affecting prognosis and personalized management plans, therefore it is an obstacle to produce valid results and conclusions on a specific comorbidity.

To address these issues, prospective multicenter registries should be established to track SA cases across diverse demographics. Additionally, meta-analyses and case-control studies should be conducted to enhance data reliability and identify key trends (101). Moreover, Real-world evidence (RWE) databases and integrated health records can be valuable in collecting robust data for studies due to their ability to provide longitudinal data, leading to more comprehensive patient histories. Additionally, RWE databases facilitate large-scale data collection by incorporating information from diverse populations, enhancing the generalizability and reliability of research findings (102). Furthermore, some novel therapies that could be explored for SA in high-risk populations are categorized into targeted biologics and biomarkers. An example of novel targeted biologics entails tocilizumab, an IL-6 inhibitor that could help reduce excessive inflammation arising from SA (103). Additionally, secukinumab, an IL-17 inhibitor currently used for psoriatic arthritis, could potentially prevent joint destruction in SA by blocking IL-17-mediated inflammation (104). Moreover, biomarkers play a vital role in risk stratification and personalized treatment approaches. For instance, C-reactive protein (CRP) levels serve as an indicator of systemic inflammation and can be useful for monitoring treatment response (105).

Lastly, given the prognostic value of the CCI in SA, its role in initial clinical assessment warrants further investigation. Determining whether it should be used routinely, or even mandatorily, requires stronger evidence and clearer clinical guidelines. Future studies should address this gap.

## 4 Conclusion

Comorbidities play a critical role in shaping the management and prognosis of patients with SA, significantly influencing the risk of complications, treatment effectiveness, and long-term outcomes. Conditions such as diabetes, cardiovascular disease, and obesity can impair immune responses, delay diagnosis, and complicate therapeutic interventions, ultimately contributing to higher morbidity and mortality rates. As such, a comprehensive approach to patient care that addresses these underlying health issues is essential for improving outcomes in SA. Future research should focus on refining management strategies that consider both the infectious and comorbid factors, with an emphasis on early detection, personalized treatment, and targeted interventions to optimize recovery and reduce the burden of this potentially life-threatening or invalidating condition.
